# Biomechanical evaluation of a novel hockey-stick locking plate featuring a pes anserinus-sparing design: a finite element analysis

**DOI:** 10.3389/fsurg.2025.1722354

**Published:** 2026-01-15

**Authors:** Xiao Wang, Haohua Lai, Songyan Zhang, Zhiheng Tu, Zhaowei Yin, Gadisa Musa Wako, Junwei Yan, Bin Liang

**Affiliations:** 1 Department of Orthopaedics, Nanjing First Hospital, Nanjing Medical University, Nanjing, China; 2 Department of Orthopaedics, Nanjing Qixia District Hospital, Nanjing, China; 3 Department of General Surgery, Nanjing First Hospital, Nanjing Medical University, Nanjing, China

**Keywords:** biomechanics, finite element analysis, novel hockey-stick locking plate, Schatzker classification, tibial plateau fracture

## Abstract

**Background:**

Surgical fixation for Schatzker IV tibial plateau fractures presents a clinical dilemma: achieving robust stability while avoiding impingement on the pes anserinus tendons. This study evaluated the biomechanical profile of a novel hockey-stick locking plate (NHLP), which is anatomically contoured to address this challenge by being placed anteriorly.

**Methods:**

A finite element model of a standardized Schatzker IV fracture was created. Three fixation methods were simulated: the novel hockey-stick locking plate (NHLP), the traditional T-shaped locking plate (TTLP), and the double reconstruction locking plates (DRLP). The models were subjected to four loading conditions: three physiological loads, a low axial load (500 N), a moderate combined load (1,500 N axial compression plus 150 N anterior shear force), and a high axial load (2,500 N) and a fourth “worst-case” load scenario combining a 1,700 N axial force, a 200 N anterior shear force, and a 10° varus tilt. Key biomechanical metrics, including implant stress, construct stability, fragment displacement, fracture interface mechanics and fatigue safety factor, were analyzed.

**Results:**

Under physiological loading, the NHLP construct demonstrated the lowest peak von Mises stress on the implant. At the high axial load of 2,500 N, the peak stress on the NHLP (159.8 MPa) was 15% lower than that on the TTLP (188.1 MPa) and 35% lower than that on the DRLP (245.5 MPa). In the “worst-case” scenario, all constructs exhibited high safety factors. In terms of stability, the NHLP provided displacement comparable to that of the TTLP, and both were substantially more stable than the DRLP construct, which exhibited the largest displacement under high load. Paradoxically, the DRLP construct consistently resulted in the highest degree of implant stress and the least stability. At the fracture interface, the NHLP maintained a stable environment across all loads, with key metrics remaining within a range conducive to bone healing.

**Conclusion:**

This finite element analysis demonstrated that the NHLP provides fracture stability while reducing peak implant stress under physiological loading. These findings support the biomechanical feasibility of its pes anserinus-sparing design, providing a strong rationale for further investigation.

## 1 Introduction

Tibial plateau fractures, particularly Schatzker IV fractures involving the medial plateau, present a significant treatment challenge, carrying risks of varus collapse, articular incongruity, and subsequent post-traumatic osteoarthritis (1, 2). Anatomical reduction and stable fixation are essential to permit early joint mobilization and optimize outcomes (3, 4). However, the high compressive loads across the medial tibial plateau demand robust implant support, making the choice of fixation strategy critical.

Existing implants for the medial tibia, such as T-shaped or reconstruction plates, present a well-documented compromise between mechanical strength and soft-tissue compatibility. Their profiles are often poorly adapted to the complex three-dimensional contour of the anteromedial tibial flare, leading to hardware prominence over the pes anserinus (5–7). This implant-tendon conflict is a significant cause of postoperative pain and may necessitate secondary surgery for hardware removal (8, 9). While attempts to mitigate this issue with smaller or manually bent plates may reduce irritation, they potentially sacrifice the mechanical stability required to resist physiological varus forces.

**Figure 1:**
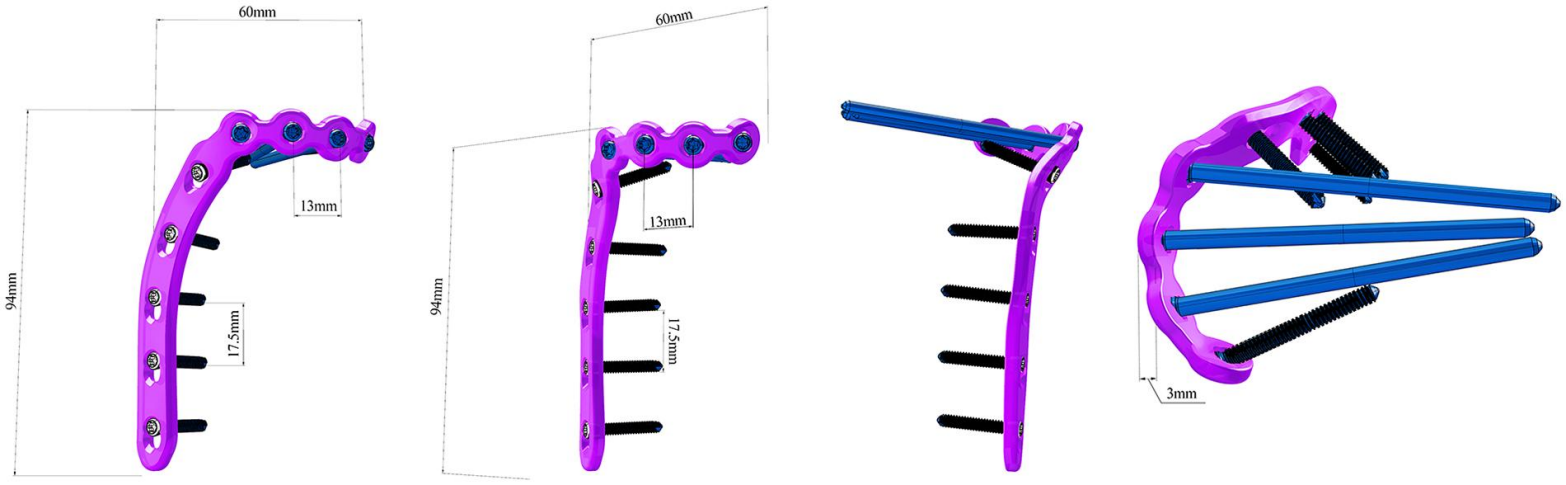
Design features and specifications of the novel hockey-stick locking plate (NHLP). The figure displays key dimensions of the NHLP (including screw spacing, plate thickness, and overall length/width) alongside views from multiple angles that illustrate its anatomical profile. The plate is engineered to match the complex contour of the medial proximal tibia, with a design comprising three integrated sections: a distal straight segment with combi-holes, a crescent-shaped turning segment to follow the tibial flare, and a proximal head with multiplanar curves and universal locking holes. These holes permit the insertion of 3.5 mm locking screws at a variable angle of up to 30° from the central axis for multidirectional subchondral support. Material specifications: The implant is made of Ti-6Al-4V ELI (Grade 23) alloy with a surface anodization treatment. The undersurface of the plate is flat.

**Figure 2:**
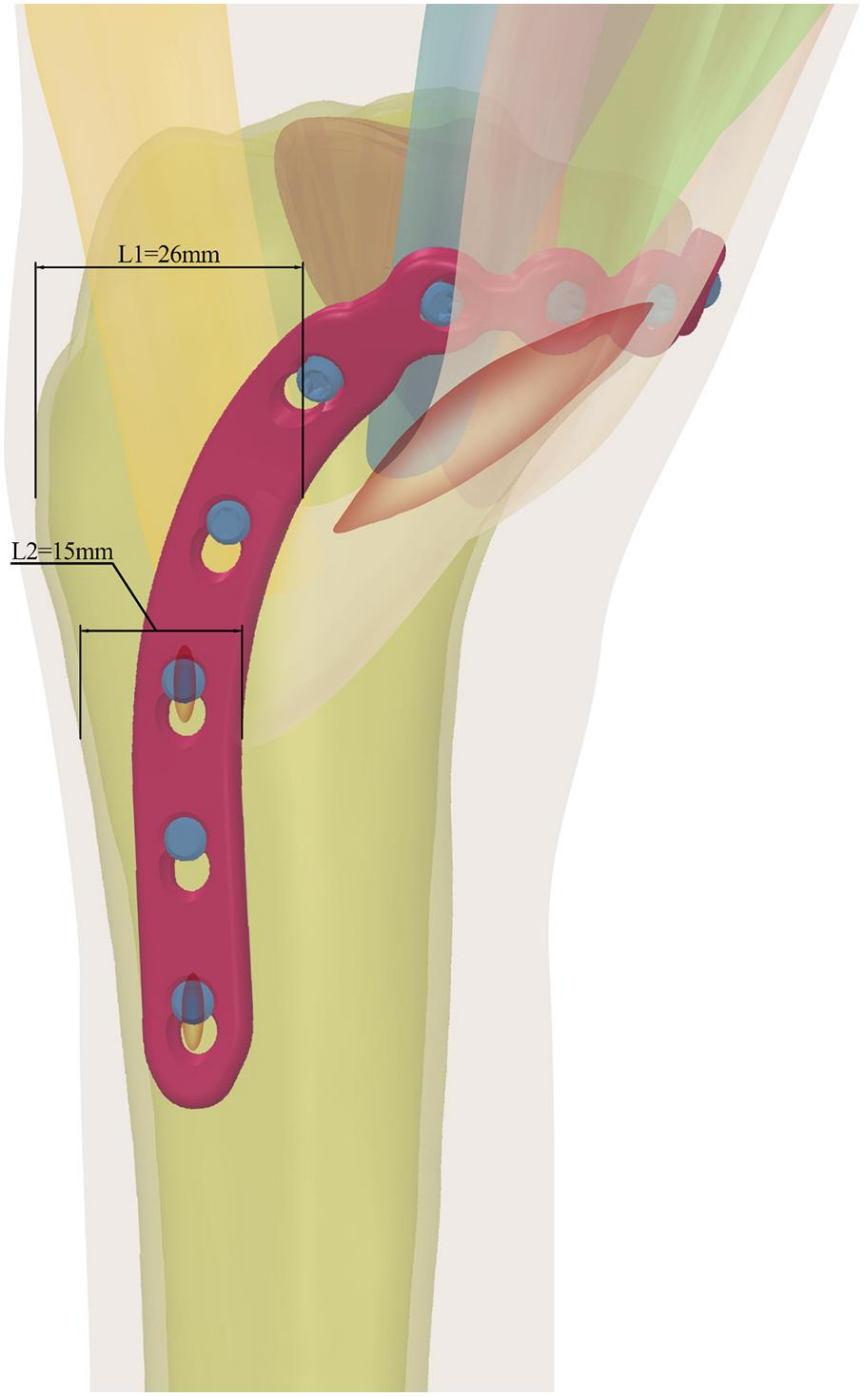
Anatomical placement and pes anserinus-sparing principle of the novel hockey-stick locking plate (NHLP). The NHLP was conceived from an anatomy-driven design philosophy. As illustrated, its trajectory maintains a safe distance from critical soft tissue structures, positioning the plate anterior to the pes anserinus insertion zone. This placement is quantified by the distances from the plate's posterior edge to the anterior tibial crest (L1 = 26 mm) and to the inferior insertion point of the pes anserinus (L2 = 15 mm). The corresponding surgical incision, which benefits from this anterior placement, is also indicated.

To address this dilemma, we designed a novel hockey-stick locking plate (NHLP) with an anatomically contoured profile intended to reduce soft-tissue impingement. The plate geometry features a crescent-like turning segment and a multiplanar proximal curve designed to fit the tibial flare while offering versatile screw options for multidirectional stability (Figure 1). This anatomical contouring is specifically intended to follow the tibial flare and avoid prominent hardware over the pes anserinus insertion site, thereby minimizing potential soft-tissue impingement (Figure 2). Before clinical consideration, its biomechanical competence must be rigorously established. Therefore, this finite element study aimed to evaluate the biomechanical performance of the NHLP construct in comparison to a traditional T-shaped locking plate (TTLP) and a double reconstruction locking plate (DRLP) on a standardized Schatzker IV fracture model. We hypothesized that under physiological loading, the NHLP construct would maintain fracture-site motion and implant stresses within clinically acceptable thresholds known to be permissive for bone healing, thereby confirming its biomechanical viability as a soft-tissue-sparing alternative.

## 2 Methods

### 2.1 Finite element model development

**Figure 3:**
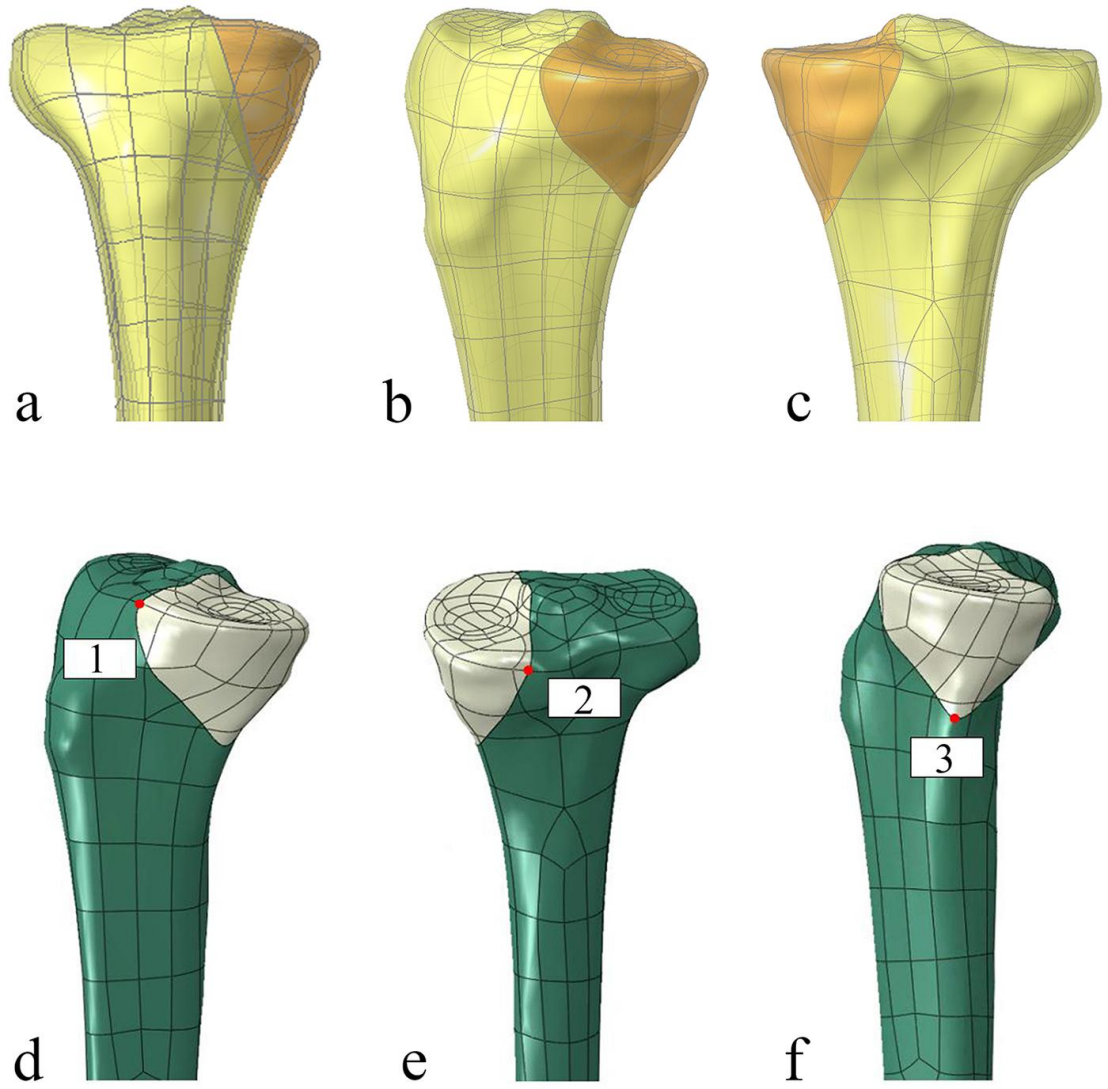
Finite element model of the Schatzker IV tibial plateau fracture and measurement setup. **(a–c)** The fracture model creation process, illustrating the standardized fracture lines based on CT data. **(d–f)** The three measurement points on the articular surface used for displacement analysis.

A three-dimensional (3D) solid model of the right tibia was developed from a computed tomography (CT) scan of a healthy 32-year-old male volunteer (height: 178 cm; weight: 75 kg) with no history of lower limb pathology. The scan was acquired using a 128-slice spiral CT scanner (Philips CT 6000, Netherlands) at 120 kV and 150 mA with a 0.5 mm slice thickness. The resulting DICOM data were imported into Mimics 20.0 (Materialise, Belgium) for image segmentation and 3D reconstruction of the tibial geometry. The model was subsequently exported to Creo 7.0 (PTC, USA) for geometric processing. A standardized Schatzker IV medial tibial plateau fracture was simulated by creating an oblique osteotomy, as described by Cift et al. (10). This osteotomy originated 5 cm distal to the medial joint line and extended superiorly to the medial intercondylar eminence (Figure 3), creating a fracture model consistent with previous finite element studies on this topic (11).

**Figure 4:**
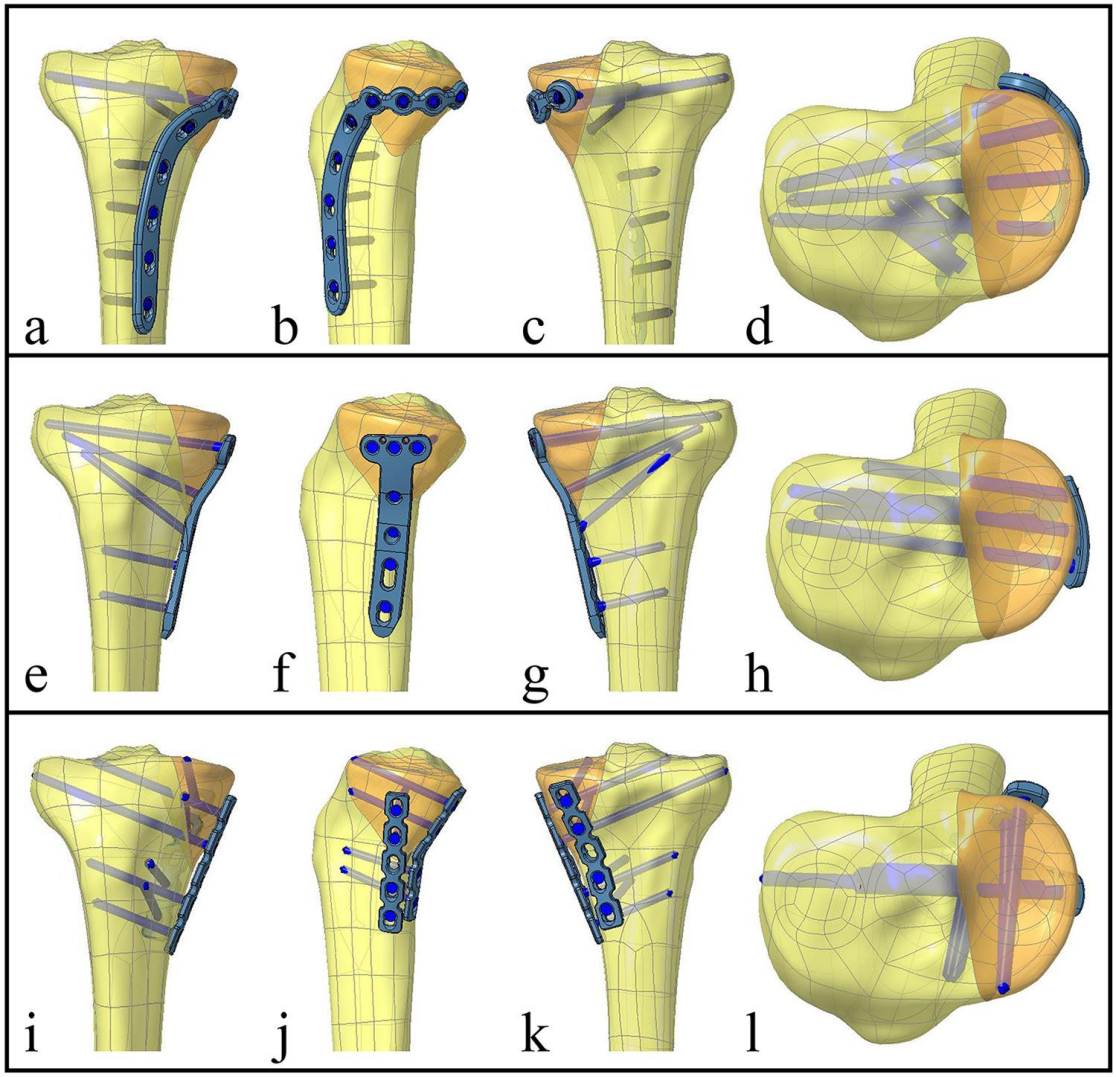
Final assembly of the three internal fixation constructs for the schatzker type IV tibial plateau fracture model. **(a–d)** Novel hockey-stick locking plate (NHLP) construct. **(e–h)** Traditional T-shaped locking plate (TTLP) construct. **(i–l)** Double reconstruction locking plates (DRLP) construct.

A simulation of virtual implantation was performed for the three fixation constructs (NHLP, TTLP, and DRLP) on the fractured tibia model (Figure 4). All the implants were modeled for use with 3.5 mm screws. Boolean operations were utilized in Creo 7.0 to precisely model the screw holes and ensure a conforming, interference-free interface between the implants and the bone. The finalized assembly models were then exported in STEP format for analysis.

### 2.2 Material properties, interactions, and boundary conditions

All materials in the model were defined as homogeneous, isotropic, and linearly elastic, a standard simplification in comparative biomechanical finite element analysis (FEA) studies. The material properties of the cortical bone, cancellous bone, and titanium alloy (Ti-6Al-4V) implants were assigned on the basis of values reported in the established literature (12, 13) and are detailed in Table 1.

**Table 1:** Parameters of the finite element models.

Material	Elastic modulus, MPa	Poisson's ratio
Cortical bone	16,800	0.30
Cancellous bone	840	0.20
Internal fixation	1,10,000	0.35

Interactions between components were defined to simulate physiological conditions. A surface-to-surface frictional contact was established between the fracture fragments, with a friction coefficient of 0.5 (14). A friction coefficient of 0.3 was assigned to the interface between the plate and the bone (15). To replicate the rigid fixation of a locking screw construct, a “bonded” constraint was applied to the screw-plate and screw-bone interfaces, prohibiting relative motion. The distal end of the tibia was fully constrained in all six degrees of freedom to simulate a fixed ankle joint.

### 2.3 Meshing and loading conditions

The models were imported into Abaqus 2020 for meshing and analysis. All the components were meshed using high-order ten-node quadratic tetrahedral elements (C3D10). To ensure results were independent of mesh density, a rigorous mesh convergence study was performed. As detailed in Table 2, this analysis confirmed that the peak implant stress stabilized and met the pre-defined <5% convergence criterion for mesh sizes of 2.0 mm and finer. Therefore, based on these findings and to balance accuracy with computational cost, a hybrid meshing strategy was adopted: a fine mesh of 1.0 mm for the implants and adjacent bone regions, and a coarser mesh of 3.0 mm for the remaining tibial shaft. This resulted in final models with total element counts ranging from approximately 250,000 to 350,000 and negligible analysis warnings for element distortion (<1%).

**Table 2:** Mesh convergence study results for the DRLP construct under 500 N axial load.

Mesh size (mm)	Implant element count	Implant node count	Peak von Mises stress (MPa)	% Change from previous	Overall tibial displacement (mm)
3.0	16,552	30,011	40.5	—	0.896
2.5	17,735	32,242	40.9	+0.99%	0.896
2.0	19,933	36,276	45.4	+11.0%	0.896
1.5	28,622	50,617	44.9	−1.10%	0.896
1.0	61,852	102,117	45.6	+1.56%	0.896

Convergence was considered achieved when the percentage change in peak stress between consecutive refinements was <5%.

**Figure 5:**
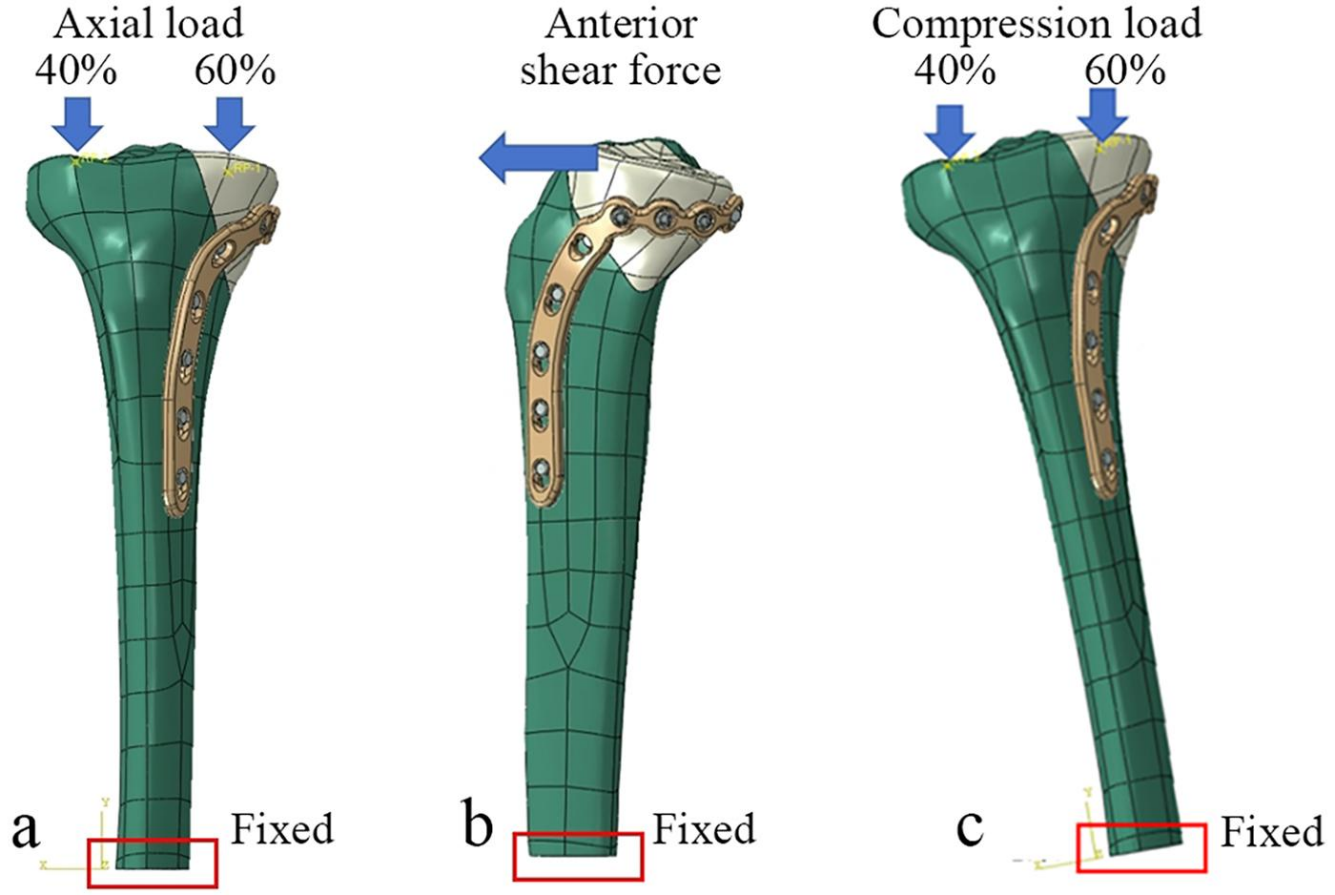
Schematic of the loading conditions applied to the finite element model. **(a)** An axial compressive force was applied to the medial and lateral tibial plateaus. **(b)** An anterior shear force was applied to the tibial plateau. **(c)** The “worst-case” scenario, which simulated a 10° varus knee moment by applying a corresponding valgus tilt to the tibia, in combination with axial and shear forces.

Four loading scenarios were defined to simulate a range of physiological activities (Figure 5). The first three scenarios represented a range of physiological activities: (1) Light load (500 N axial), (2) Moderate load (1,500 N axial + 150 N anterior shear), and (3) High load (2,500 N axial). In these scenarios, the axial force was distributed 60% medially and 40% laterally, consistent with gait kinetics data (16). A fourth, “worst-case load” scenario was added based on gait analysis data to assess performance under more challenging conditions: a combined 1,700 N axial force, a 200 N anteroposterior shear force, and a 10° varus tilt (17–20). The loads were applied via two reference points. Each reference point was coupled to the articular surface of the corresponding tibial condyle (medial or lateral) to simulate joint reaction forces. The anterior shear force was included to replicate the complex mechanical environment during dynamic movements (11, 21–23).

### 2.4 Evaluation metrics

The biomechanical performance of the three constructs was compared using several key metrics. The von Mises stress (VMS) distribution on the implants was analyzed to identify areas of stress concentration. To quantitatively assess the risk of implant failure under high-stress conditions, a fatigue safety factor (FSF) was calculated for both the high Load and “worst-case load” scenarios. The FSF was defined as the ratio of the implant material's yield strength (*σ*y) to the peak von Mises stress (*σ*vM) observed in the implant (FSF = *σ*y/σvM). At the fracture site, the maximum principal strain within the cancellous bone was evaluated, as interfragmentary strain is a critical stimulus for bone healing. To assess the overall structural stability, the maximum displacement of the main fracture fragment was measured. Finally, the relative displacement between the fracture surfaces was quantified to evaluate interfragmentary motion under load.

## 3 Results

### 3.1 Implant stress and overall construct stability

The biomechanical results for the three fixation constructs under all four loading conditions are summarized in Table 3.

**Table 3:** Key biomechanical results for all constructs under all loading conditions.

Loading condition	Internal fixation	Peak implant VMS (MPa)	Max fragment displacement (mm)	Peak tibial VMS (MPa)	Peak interfacial VMS (MPa)	Peak interfacial strain	Interfragmentary gap (Point 1/2/3, mm)	FSF
Light loading, 500 N	NHLP	30.4	0.849	14.4	4.21	0.0012	0.009/0.007/0.013	—
	TTLP	37.2	0.835	14.4	2.74	0.0013	0.006/0.006/0.005	—
	DRLP	45.6	0.878	21.7	4.35	0.0028	0.011/0.016/0.007	—
Moderate combined loading, 1,500 N axial + 150 N shear	NHLP	76.0	1.492	33.7	10.82	0.0035	0.029/0.016/0.037	—
	TTLP	112.3	1.460	52.9	6.98	0.0032	0.022/0.011/0.017	—
	DRLP	106.0	1.540	63.3	10.66	0.0052	0.031/0.028/0.022	—
High loading, 2,500 N axial	NHLP	159.8	4.854	81.0	20.22	0.0062	0.047/0.037/0.065	5.44
	TTLP	188.1	4.811	80.9	13.93	0.0056	0.035/0.033/0.023	4.63
	DRLP	245.5	5.062	111.5	22.95	0.0159	0.062/0.090/0.036	3.54
Worst-case loading, 1,700 N axial + 200 N shear + 10° varus	NHLP	50.5	5.430	73.3	11.54	0.0035	0.026/0.014/0.038	17.23
	TTLP	62.8	5.680	73.5	9.03	0.0031	0.020/0.012/0.014	13.85
	DRLP	114.4	5.701	73.5	10.83	0.0040	0.029/0.024/0.020	7.60

NHLP, novel hockey-stick locking plate; TTLP, traditional t-shaped locking plate; DRLP, double reconstruction locking plate; FSF, fatigue safety factor; VMS, von Mises stress. The FSF was calculated for the high load and worst-case scenarios only. A “—” indicates a non-applicable value.

Under physiological axial loads, the NHLP construct consistently registered the lowest peak VMS on the implant. Under the high-load condition of 2,500 N, the peak VMS on the NHLP was 159.8 MPa, which was 15% lower than the 188.1 MPa observed in the TTLP construct and 35% lower than the 245.5 MPa in the DRLP construct. Regarding overall stability, the maximum displacement of the main fracture fragment was similar for the NHLP and TTLP constructs under these loads, while the DRLP construct showed markedly greater displacement.

**Figure 6:**
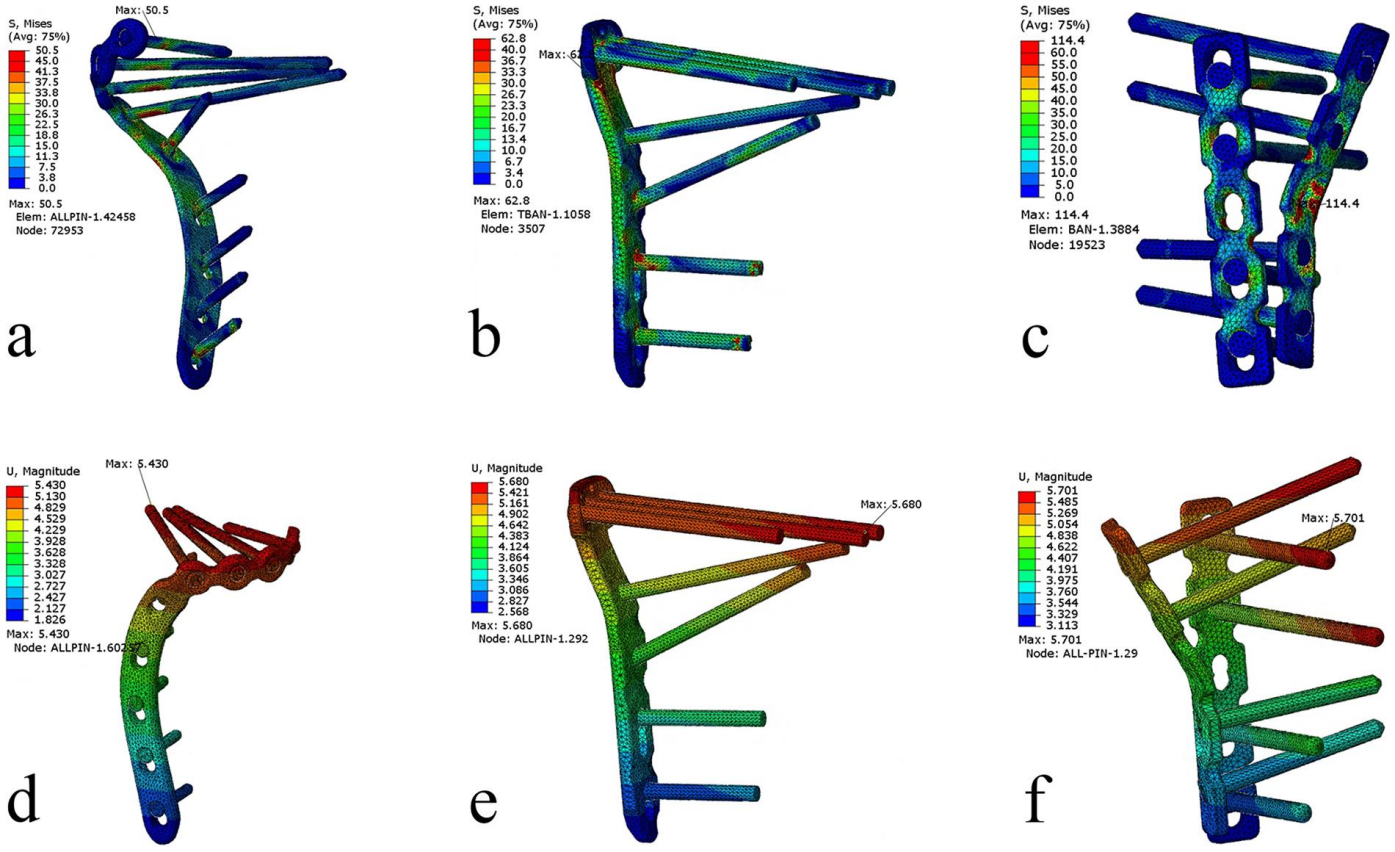
Von Mises stress (VMS) distributions and displacement magnitudes for the three internal fixation constructs under the “worst-case” loading scenario. **(a–c)** VMS distributions for the novel hockey-stick locking plate (NHLP), traditional T-shaped locking plate (TTLP), and double reconstruction locking plates (DRLP), respectively. **(d–f)** The corresponding displacement magnitudes and distributions for each construct.

Under the “worst-case load” scenario, the peak implant VMS for the NHLP was 50.5 MPa, compared to 62.8 MPa for the TTLP and 114.4 MPa for the DRLP. The corresponding maximum fragment displacements were 5.430 mm, 5.680 mm, and 5.701 mm, respectively, which were the highest displacements recorded across all loading scenarios. The stress distribution patterns under this condition are visualized in Figure 6.

The calculated FSF under the “worst-case load” was 17.23 for the NHLP, 13.85 for the TTLP, and 7.60 for the DRLP. For the high load condition, the FSF values were 5.44, 4.63, and 3.54 for the NHLP, TTLP, and DRLP, respectively.

### 3.2 Stress distribution within the tibia

Analysis of stress within the tibia showed that under physiological axial loads, the DRLP construct generally produced the highest peak VMS. However, under the “worst-case load”, peak tibial stresses were similar across all three groups: 73.3 MPa for the NHLP, and 73.5 MPa for both the TTLP and DRLP constructs (Table 3).

### 3.3 Fracture interface mechanics

**Figure 7:**
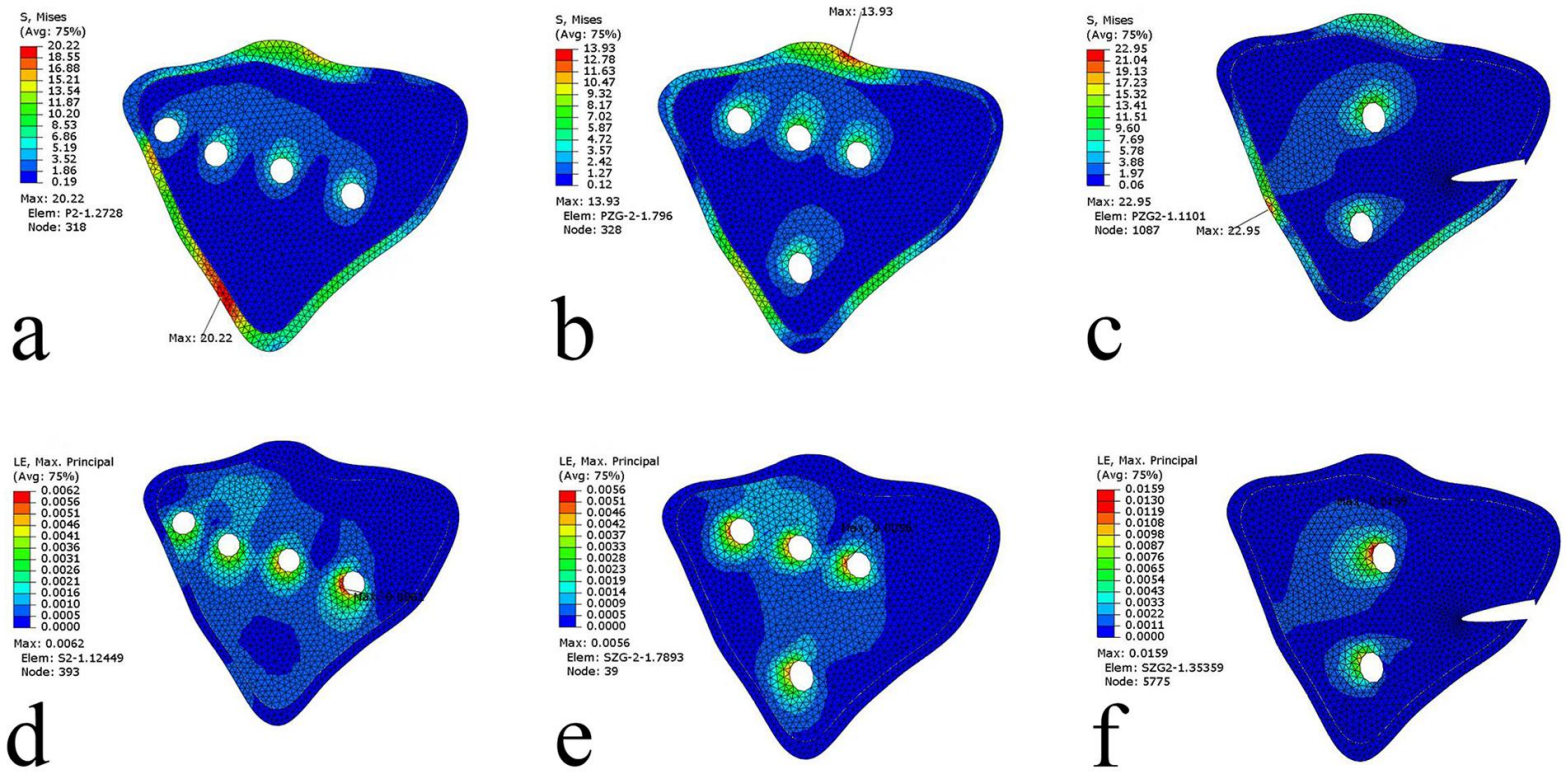
Stress and strain distributions across the fracture interface under the 2,500 N high loading condition. **(a–c)** Von Mises stress (VMS) distributions for the novel hockey-stick locking plate (NHLP), traditional T-shaped locking plate (TTLP), and double reconstruction locking plates (DRLP), respectively. **(d–f)** The corresponding maximum principal strain distributions for each construct.

The mechanical environment at the fracture interface is detailed in Table 3. The maximum principal strain under the high load condition was 0.0062 for the NHLP, 0.0056 for the TTLP, and 0.0159 for the DRLP, with distribution patterns visualized in Figure 7.

Regarding interfragmentary motion, the TTLP construct demonstrated the highest stability under both high load and “worst-case” conditions. For example, at the critical lateral measurement point (Point 3), the gap displacement under high load was smallest for the TTLP, and this advantage was maintained under the “worst-case load” (0.0140 mm for TTLP vs. 0.0381 mm for NHLP). The locations of these measurement points are illustrated in Figure 3.

## 4 Discussion

The surgical management of Schatzker IV tibial plateau fractures often presents a clinical dilemma: achieving the robust fixation necessary for joint congruity while minimizing disruption to the vital soft-tissue structures of the medial knee, particularly the pes anserinus (3, 4, 24). Conventional plating techniques can lead to hardware prominence and tendon irritation, potentially compromising rehabilitation (5–7). The NHLP was conceived from an anatomy-driven design philosophy to address this specific challenge. Its design maintains a trajectory of 15–26 mm from the anterior tibial crest, which, when compared to anatomical data, places the plate anterior to the pes anserinus insertion zone (Figure 2) (25). This study, therefore, evaluates the biomechanical viability of this anatomy-conscious design.

The biomechanical performance of the NHLP appears to support the viability of its design. A primary finding under physiological loading was that the NHLP construct consistently exhibited the lowest peak VMS on the implant. For instance, under the 2,500 N high load, the peak VMS was 159.8 MPa, resulting in a superior FSF of 5.44 (Table 3). This outcome is likely a direct consequence of its geometry. The plate's multiplanar curvature, engineered to match the tibial contour, may facilitate a more efficient load transfer from the bone, thereby minimizing the stress risers that can occur at the sharp geometric transitions of plates with a more standardized shape. The elongated distal segment of the NHLP, while creating a longer lever arm was created, did not result in greater peak implant stress. It is hypothesized that the multiple points of fixation along this segment create a composite bone-implant structure that distributes the load, rather than concentrating it at a single fulcrum. In the context of repetitive physiological loading, a lower peak operational stress is a fundamental factor in mitigating the risk of fatigue-related material failure (26). The introduction of a varus moment in the “worst-case” scenario presented a different biomechanical environment. This condition led to a general decrease in implant stress for all constructs, as more load was transferred through the bone. Consequently, the calculated FSF for all three methods was markedly high, suggesting a low risk of acute implant failure under this specific challenge. In terms of stability, the NHLP demonstrated the smallest fragment displacement (5.430 mm), performing comparably to the TTLP (5.680 mm).

The performance of the DRLP construct, however, highlights that biomechanical stability is not merely a function of implant quantity, but of how effectively load is managed across the entire construct. In any fixed fracture, axial load is shared among several parallel pathways. A portion of the load travels through the implant itself, passing from the proximal screws into the plate. Another portion is transmitted directly across the fracture interface where bone-to-bone contact is present. Furthermore, a third pathway exists where load is transferred directly from the fracture fragment into the tibial metaphysis through the subchondral screws, which bridge the fracture line and engage the stable, underlying bone. The DRLP construct, despite using two plates, exhibited the highest implant stress of 245.5 MPa under high physiological load and inferior overall stability (Table 3). This paradoxical result suggests a failure in efficiently distributing the load among these pathways. With only one subchondral screw on each of its two non-synergistic plates, the construct is heavily reliant on the implant pathway. This overwhelms these two mechanically weak points, leading to high stress concentrations (Figure 6) and greater fragment displacement, as the construct fails to effectively utilize both direct bone-to-bone contact and the screw-bone bridging pathway for load sharing. In contrast, the multi-screw “raft” configurations of the TTLP and NHLP provide a more robust initial support, allowing for a more balanced load distribution across all available pathways.

The mechanical environment at the fracture interface must be interpreted within the broader context of overall stability and implant safety. Under high loading, the peak principal strains were 0.56% for the TTLP, 0.62% for the NHLP, and 1.59% for the DRLP (Table 3). Given that all these values are well below the 2% strain level typically associated with robust secondary callus formation, all three constructs created a highly stable environment favoring primary bone healing (27, 28). However, within this favorable healing context, the NHLP showed significantly lower implant stress, evidenced by a peak VMS of 159.8 MPa compared to the TTLP's 188.1 MPa (Table 3).

From a clinical standpoint, these biomechanical findings are significant as they validate the feasibility of an anatomy-conscious design. The primary impetus for the NHLP's design was not to achieve superior mechanical strength, but to respect the medial soft-tissue envelope by positioning the plate anterior to the pes anserinus. This design philosophy is an evolution of our previous clinical experience, where standard plates were manually contoured intraoperatively to achieve a similar anatomy-sparing shape (29). The central question of this study was whether a pre-contoured implant based on this principle could provide sufficient stability, or if its “cantilever-like” design would come at an unacceptable mechanical cost.

Our data answers this question favorably. The results demonstrate that the NHLP provides a level of stability comparable to established fixation methods, effectively dispelling concerns about its mechanical viability. Unexpectedly, the design also exhibited a distinct mechanical advantage: the significantly lower operational stress under physiological loading suggests a reduced long-term risk of fatigue failure. This finding is critical, as it indicates that the goal of soft-tissue preservation does not have to compromise, and may even enhance, the implant's mechanical longevity. Furthermore, the multidirectional proximal locking screws provide the versatility to create a subchondral “raft,” a well-established strategy for resisting articular subsidence (30–34). Therefore, this study confirms that the NHLP is not only a mechanically sound option but one that successfully harmonizes the principles of soft-tissue respect with robust biomechanical performance, providing a strong rationale for its continued clinical investigation.

Several limitations of this study should be acknowledged. First, the findings are based on a single computational model derived from the CT data of a healthy young male. This significantly impacts the generalizability of the results, particularly regarding bone quality. The observed biomechanical advantages of the NHLP, such as its lower operational stress compared to other constructs, are contingent on the robust bone stock of this model. It is highly plausible that in an osteoporotic bone model, where bone provides less support, a greater portion of the load would be transferred to the implants, potentially diminishing or even eliminating this stress differential. Therefore, the performance of these constructs in an elderly population remains an open and critical question. Second, the loading conditions were static. This approach was intentionally chosen as the role of FEA in this stage of development is to serve as an initial, cost-effective evaluation of the design's fundamental mechanical viability. If a construct fails to demonstrate adequate performance under static peak loads, proceeding to more resource-intensive cyclic testing, either computationally or on cadaveric models, would be unwarranted. However, it must be acknowledged that this static approach omits the effects of cyclic loading, which are crucial for predicting long-term phenomena such as fatigue-related material failure and progressive screw loosening. Consequently, while our FSF calculations provide a useful proxy for fatigue resistance, a comprehensive fatigue life assessment remains a task for future mechanical testing. Finally, as previously mentioned, the model's exclusion of soft tissues means that the anticipated clinical benefit of avoiding pes anserinus impingement remains a well-founded hypothesis based on anatomical positioning. These limitations collectively define the scope of this study as a foundational, preclinical validation. Future work is essential to address these points, including FEA on varied bone geometries and densities, cadaveric mechanical testing to investigate cyclic performance, and ultimately, prospective clinical trials to confirm the NHLP's real-world efficacy and safety.

## 5 Conclusions

In this FEA, the NHLP maintained fracture displacement and interfragmentary strain within accepted thresholds for stability in a Schatzker IV tibial plateau fracture model. Under identical loading conditions, the NHLP resulted in a lower peak VMS on the implant than did the TTLP and DRLP. While these computational results support the mechanical feasibility of the NHLP design, they require validation through future cadaveric testing and clinical trials.

## Data Availability

The raw data supporting the conclusions of this article will be made available by the authors, without undue reservation.
